# Comparison of Sexual Knowledge, Attitude, and Behavior between Female Chinese College Students from Urban Areas and Rural Areas: A Hidden Challenge for HIV/AIDS Control in China

**DOI:** 10.1155/2016/8175921

**Published:** 2016-12-22

**Authors:** Min Chen, Yong Liao, Jia Liu, Wenjie Fang, Nan Hong, Xiaofei Ye, Jianjun Li, Qinglong Tang, Weihua Pan, Wanqing Liao

**Affiliations:** ^1^Department of Dermatology and Venerology, Shanghai Key Laboratory of Molecular Medical Mycology, Shanghai Institute of Medical Mycology, Changzheng Hospital, Shanghai, China; ^2^Department of Dermatology and Venerology, PLA Army General Hospital, Beijing, China; ^3^Department of Health Statistics, Second Military Medical University, Shanghai, China; ^4^Medical Clinic of General Political Department of PLA, Beijing, China

## Abstract

Currently, research in sexual behavior and awareness in female Chinese college students (FCCSs) is limited, particularly regarding the difference and the influencing factors between students from rural areas and urban areas. To fill the gap in available data, a cross-sectional study using anonymous questionnaires was conducted among 3193 female students from six universities located in Beijing, Shanghai, and Guangzhou, China, from February to June, 2013. Of the 2669 respondents, 20.6% and 20.9% of the students from urban and rural areas, respectively, reported being sexually experienced. The proportion of students who received safe-sex education prior to entering university from rural areas (22.4%, 134/598) was lower (*P* < 0.0001) than the proportion from urban areas (41.8%, 865/2071). Sexual behavior has become increasingly common among FCCSs, including high-risk sexual behavior such as unprotected commercial sex. However, knowledge concerning human immunodeficiency virus (HIV)/acquired immune deficiency syndrome (AIDS) transmission and the risks is insufficient, particularly for those from rural areas, which is a challenge for HIV/AIDS control in China. The Chinese government should establish more specific HIV/AIDS prevention policies for Chinese young women, strengthen sex education, and continue to perform relevant research.

## 1. Introduction

China has been divided into a dual-structure society for decades, where those living in rural areas (approximately 50.3% of the population) typically have lower-quality educations and lower-income jobs than those living in urban areas [1–3]. Dating from the 1980s, China has experienced dramatic social and cultural changes due to the open-door policy and economic reforms. Chinese attitude and behavior regarding sex have also profoundly changed, including topics such as premarital sex, extramarital sex, and homosexual behavior, especially in the younger generation [4–7].

Although the human immunodeficiency virus (HIV) prevalence remains relatively low (<0.1% among adults), the HIV epidemic in China is spreading from the traditional high-risk population to some in the general population [5–9]. In 2005, an estimated half of new HIV infections in China occurred via unprotected heterosexual intercourse [10]. The experience in African countries has already indicated that the HIV epidemic grows rapidly, when heterosexual transmission becomes the main mode of HIV transmission [11].

In 2013, the number of enrolled female Chinese college students (FCCSs) reached approximately 8.9 million in China [2]. A disproportionate increase in the incidences of new HIV infections has recently been noted among 15–24 year olds, compared to other population segments in many countries including China [11]. Statistics from the Chinese Center for Disease Control and Prevention (China CDC) revealed that new HIV infections amongst young students increased from 0.96% of all cases in 2006 to 1.73% in 2011 [9]. At the same time, an increasing proportion of FCCSs is participating in sexual behavior, including premarital sex and other high-risk heterosexual intercourse, such as multiple-partner sex and/or commercial sex [7, 12, 13].

Thus, concern is growing regarding sexual knowledge, attitude, and behavior among FCCSs, who are vulnerable to HIV infection due to gender inequality and deficient sex education [12–17]. Although previous studies have yielded useful information on sexual behavior in FCCSs [12, 13, 15], research in this field is still limited, particularly regarding the difference and the influencing factors between students from rural and urban areas [7]. The study objectives were to comparatively analyze the differences in sexual knowledge, attitudes, and behavior of FCCSs from urban and rural areas and to explore the factors associated with different sexual profiles between these two groups.

## 2. Methods

### 2.1. Study Population

The study was a school-based survey using a self-administered questionnaire for FCCSs, conducted in Beijing, Shanghai, and Guangzhou from February to June, 2013. These three cities are the most developed centers of economy, science, and culture in China and are the locations of 89, 68 and 138 universities, respectively. All universities in each city had an equal probability for selection. Two universities located in Beijing, two in Shanghai, and two in Guangzhou were recruited using stratified random sampling. After being informed about the purpose of the study, all six universities agreed to participate. Students of the six universities were informed of the purpose of the study prior to administration of the questionnaire. Sampling on each campus was purposeful to assure the adequate representation of students across the five years of college and in the two types of majors (social sciences, such as literature, politics, or history, and natural sciences, such as physics, engineering, or biology; no medicine majors were included). Additionally, the students enrolled in the six selected universities were unmarried, studying a nonmedical major and were required to provide consent.

### 2.2. Study Design

Study participation was voluntary, and participants were reminded that the survey was anonymous. The enrolled students were required to complete the questionnaires in a classroom environment within 15 minutes. When finished, the questionnaires were inserted into a locked box.

### 2.3. Survey Procedure

After receiving permission from the universities, the researchers approached the enrolled students in their classrooms. Having explained the nature of the research, the researchers distributed a self-administered questionnaire to the students. The students were informed that the survey was anonymous and were assured of the confidentiality of their responses. Written consent was obtained prior to participation. All of the approached students agreed to participate. For the purpose of data analysis, a small number (*n* = 37) of recruited seniors were excluded from the current study because they were married. Additionally, 105 questionnaires, each with more than half of the data missing, were removed from the analysis.

### 2.4. Questionnaire

The questionnaire for this study was developed based on a review of domestic and international literature. The self-administered questionnaire was divided into four broad sections as follows: (1) sociodemographic; (2) sexual attitudes; (3) sexual behaviors; and (4) knowledge related to safe-sex. Demographic items in the questionnaire included age, types of major (liberal arts or science), family style (typical parental family, single parent family, or adopted family), monthly expenditure (frugal level, <1000 RMB per month; medium level, 1000–4000 RMB per month; and upscale level, >4000 RMB per month), current accommodation status (school dormitory or off-campus apartment), relationship status (single, yes, or no), and year of study (freshmen, sophomore, junior, senior, and graduate student).

The characteristics of sexual awareness included age of first exposure to pornographic media, such as a blue movie (<15 or >15 years old), attitudes towards homosexual sex (disapprove, approve, or not concerned), attitudes towards commercial sex (disapprove, approve, or neutral), attitudes towards premarital sex (disapprove, approve, or neutral), attitudes towards extramarital sex (disapprove, approve, or neutral), and prediction of their probability of suffering from HIV/sexually transmitted diseases (STDs) (high risk, medium risk, low risk, impossible, or never considered).

For the sexual behavioral items, questions included performing sex acts after the consumption of alcohol (yes or no), age of first masturbation (<15 or >15 years old), age of first sexual intercourse (<15 or >15 years old), contraceptive method at first sexual intercourse (condom, other methods such as pills, or none), condom use at last sexual intercourse (yes or no), the last sexual partner (heterosexual, homosexual and/or bisexual, or commercial), condom use with regular sex partners (always, often, or never), condom use with casual sex partners (always, often, or never), engaged in vaginal sex with condom (yes or no), engaged in vaginal sex without condom (yes or no), engaged in oral sex with condom (yes or no), engaged in oral sex without condom (yes or no), engaged in anal sex with condom (yes or no), engaged in anal sex without condom (yes or no), involved in commercial sex behavior (yes or no), number of sexual partners during the last year (*n* = 1 or *n* ≥ 2), and ever being diagnosed with an STDs except HIV/AIDS (yes or no).

The characteristics of the knowledge related to safe-sex included recognition of all types of STD (yes or no), recognition of all transmission modes of HIV/AIDS (correct or incorrect), recognition of methods for the prevention of HIV/AIDS (correct or incorrect), recognition of the use of condoms (correct or incorrect), and receiving safe-sex education before university (correct or incorrect).

### 2.5. Data Collection

After sampling, the questionnaires were handed out and collected by trained investigators during school hours. The relevant information from the questionnaires did not appear for the enrolled students. Subsequently, the questionnaires were checked, relevant information from each questionnaire was independently extracted by two authors, and the results were checked against one another.

### 2.6. Data Analysis

The statistical package for SAS version 9.3 (SAS Institute Inc., Cary, NC, USA) was used to enter and analyze the data. The obtained data were evaluated by frequency and percentages ratios, the Chi-square (*χ* ^2^) test, nonparametric tests, and logistic regression analyses. A *P* value less than 0.05 was deemed statistically significant.

### 2.7. Ethics

The study was approved by the Medical Ethics Committee of Shanghai Changzheng Hospital (accession number: 2012SL003). All enrolled students were informed of the purpose and the methods of the study, with no advantage or disadvantage for participation or nonparticipation.

## 3. Results

### 3.1. Sociodemographic Characteristics

Of the 3193 female students included in this cross-sectional study, 524 were eliminated from the analysis due to evident invalid responses, or incomplete responses, or inconsistent responses. Thus, the valid response rate was approximately 83.6% (2669/3193). Among the 2669 included students, approximately 37.2% (993/2669) of the students came from universities in Beijing, followed by approximately 34.3% (916/2669) and 28.5% (760/2669) from universities in Shanghai and Guangzhou, respectively. The proportions of urban students compared to rural students who had already engaged in sex were different when comparing the results from Beijing, Shanghai, and Guangzhou.

**Figure 1:**
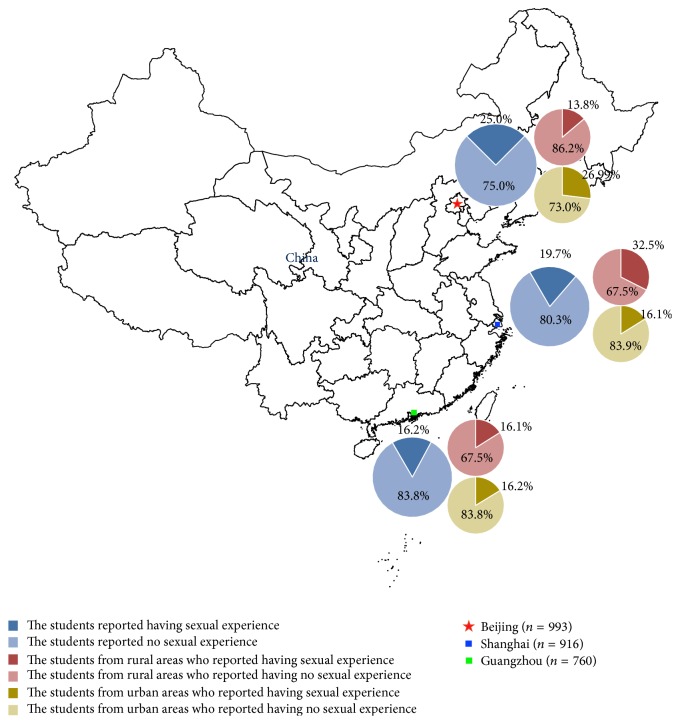
Geographic distribution and diverse characteristics of sexual behaviors of the female college students included in this study.

For all 2669 students, the mean age was 20.4 ± 1.7 years (range 17–28 years); a total of 2071 students came from urban areas, and 598 students were from rural areas in China. The distribution of their grades was relatively even, ranging from freshmen (approximately 24.3%, 648/2669) to graduate students (approximately 8.8%, 236/2669). The proportion of students from parental families in urban areas (86.9%, 1800/2071) is significantly higher (*P* < 0.0001) than those from rural areas (82.8%, 495/598). The proportion of the students living with an average monthly expenditure <1000 RMB from urban areas (58.7%, 1216/2071) was significantly lower (*P* < 0.0001) than the proportion from rural areas (79.6%; 476/598), and the proportion of students living with an average monthly expenditure of 1000–4000 RMB from urban areas (39.6%, 820/2071) was significantly higher (*P* < 0.0001) than the proportion from rural areas in China (18.6%, 111/598). The majority of the FCCSs (approximately 92.2%, 2461/2669) resided in school dormitories, but the students from urban areas (93.2%, 1930/2071) did so more frequently (*P* < 0.0001) than those from rural areas in China (88.8%, 531/598). The details are shown in Table 1 and Figure 1.

### 3.2. Sexual Attitude

Of the 2669 students, the proportion of the students from urban areas with sexual awareness prior to the age of 15 years (16.1%, 334/2071) was significantly higher than the proportion of students from rural areas in China (10.0%, 60/598). In our survey, we defined “sexual awareness” as “realize gender differences, experience sexual desire, and formed attitude towards sex.” The proportion of the students from urban areas with a tolerant attitude toward commercial sex (73.7%, 1526/2071) was significantly higher (*P* = 0.014) than the proportion of students from rural areas (78.9%, 472/598). Similar differences were also observed in the attitudes towards homosexuality (*P* < 0.001) and premarital sex (*P* = 0.001) between the students from urban and rural areas. Additionally, the proportion of students that predicted themselves to be at a high risk of suffering from HIV/AIDS infection from urban areas (1.4%, 28/2071) was significantly lower (*P* = 0.009) than that of students from rural areas in China (2.5%, 15/598). The details of sexual awareness and attitude are shown in Table 2.

### 3.3. Sexual Behavior

The proportion of FCCSs who had engaged in sex after consuming alcohol was low for the respondents (*P* = 0.851) from both the urban areas (5.9%, 122/2071) and rural areas (5.7%, 34/598). Similar results were observed in the proportion of urban and rural students for first masturbation prior to 15 years of age (*P* = 0.246), contraceptive methods used at first sexual intercourse (*P* = 0.493), condom use with casual sex partners (*P* = 0.478), and the number of sexual partners during the past year (*P* = 0.605) between the proportions of students from urban areas and rural areas. Regarding sexual patterns, vaginal intercourse using a condom was predominant and was reported by 8.0% (167/2071) of the students from urban areas and 3.8% (23/598) of the students from rural areas (*P* < 0.0001). However, vaginal intercourse without a condom was practiced by 7.0% (145/2071) of the students from urban areas and 7.4% (44/598) of the students from rural areas (*P* = 0.765). Oral sex without a condom was practiced by 5.1% (105/2071) of the students from urban areas and 6.0% (36/598) of the students from rural areas (*P* = 0.360), and anal sex without a condom was practiced by 1.7% (35/2071) of the students from urban areas and 2.3% (14/598) of the students from rural areas (*P* = 0.296). Moreover, the proportion of the students using condom at last sexual intercourse from the urban areas (44.1%, 188/426) was significantly higher (*P* < 0.0001) than the proportion of rural students (22.4%, 28/125). Notably, the proportion of students who had engaged in commercial sex as the last sexual behavior from rural areas (32.5%, 37/114) was significantly higher (*P* < 0.0001) than the proportion of students from urban areas (16.9%, 73/430) in China. Similarly, the proportion of Chinese female college students who had once been diagnosed with an STD from rural areas (11.0%, 66/598) was also higher (*P* < 0.006) than the proportion of students from urban areas (7.5%, 156/2071). The details concerning sexual behavior are shown in Table 3.

### 3.4. Sexual Knowledge and Sources of HIV/AIDS

The proportion of the students that recognized all of the transmission modes (drug use by intravenous injection, mother to child transmission, and unprotected sex) of HIV/AIDS from urban areas (30.4%, 630/2071) was significantly higher (*P* = 0.002) than the proportion of students from rural areas (23.7%, 142/598). Similar differences between these two groups were also observed concerning knowledge about all of the AIDS prevention methods. Furthermore, the proportion of Chinese female college students that received safe-sex education before entering university from rural areas (22.4%, 134/598) was significantly lower (*P* < 0.0001) than the proportion of students from urban areas (41.8%, 865/2071). Importantly, the proportion of students that correctly recognized the significance of condom use was low (*P* = 0.081) for both students from urban areas (20.1%, 416/2071) and rural areas (16.9%, 101/598). The details concerning safe-sex knowledge about STD/HIV are shown in Table 4.

**Figure 2:**
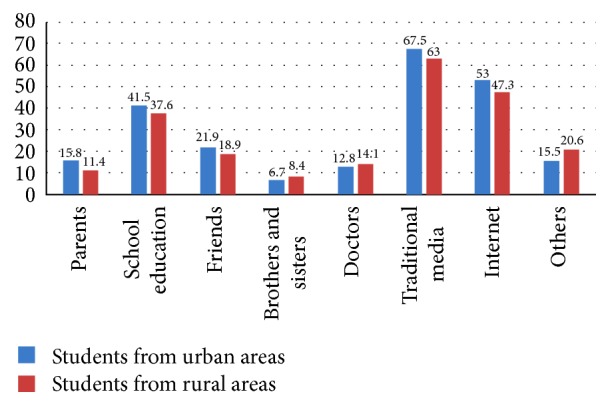
Distribution of information sources for HIV/STDs for female college students in China.

The majority of both students from urban areas (67.5%, 1367/2071) and rural areas (63%, 377/598) reported that traditional media such as newspapers or television were the most common sources of useful information for HIV/AIDS in China, followed by the Internet (53.0% compared to 47.3%), school sex education (41.5% compared to 37.6%), and friends (21.9% compared to 18.9%). The details are shown in Figure 2.

## 4. Discussion

In our study, the sociodemographic characteristics of FCCSs from urban and rural areas were diverse, which was itself a model of the dual-structure society in China. Compared to their classmates from urban areas, a higher proportion of the students from rural areas grew up in single parent or divorced families, which was partially related to their parents typically being required to obtain a higher-salary job as rural-to-urban migrant workers in Chinese cities. A previous study in Wuhan, China, suggested that divorced families were associated with earlier sex onset among FCCSs [18]. Additionally, the proportion of students from urban areas that were single was lower than that of their classmates from rural areas, but no difference was found in the rate of sexual experience between these two groups. This result suggested to us that the students from rural areas engaged in the same level of sexual activity as those from urban areas, which was in accordance with the result from a recent study in South Africa [19]. We inferred that FCCSs from rural areas might be more likely to engage in sexual behavior if they fell in love, which might also explain why a higher proportion of these students in our study preferred to live in off-campus apartments.

Our study found that FCCSs from rural areas generally maintained a more traditional attitude towards sexual intercourse regarding topics such as premarital sex than those from urban areas. These findings are a reflection of the more traditional attitude towards sex in China's rural areas and are also in accordance with the results of the previous study in China [16]. We were not surprised to see these data because students from urban areas were more likely to come into contact with Western culture than those from rural areas [17]. Remarkably, we found that a significantly higher proportion of sexually active students from China's rural areas predicted themselves to be at high risk for HIV/AIDS and/or STD infections than those from urban areas. Indeed, the rate of the students from rural areas diagnosed with STDs was higher than that of their classmates from urban areas in our study. FCCSs, particularly those from rural areas, have been the elite group of female youth in China. However, their level of mastery of safe-sex is worrying for HIV/AIDS prevention among female youth in China. The insufficient sex education provided in China, particularly in rural areas, might contribute to the higher proportion of unprotected sexual behavior and greater concerns about HIV/STDs in students from rural areas. We could draw the conclusion that no obvious advancement in HIV/AIDS prevention knowledge was observed in our study compared to previous studies [20, 21] and that prevention knowledge was poorer than that observed in Western countries such as the United States [22] or Ireland [21]. A lack of appropriate sexual knowledge will encourage students to seek sexual information from informal channels, such as television or the Internet, which was shown to play an important role in transmitting knowledge concerning HIV/AIDS in our survey and previous studies [23, 24]. However, some of the information about safe-sex in the Chinese traditional media and on the Internet is incorrect or inappropriate. Thus, we suggest that the Chinese government should focus on improving the policy of sex education in universities, secondary schools, and primary schools, which has lagged behind sexual activity among Chinese students [17, 25, 26].

Although the overall rate of sexual experience for FCCSs was still lower than in Western countries, such as the United States (approximately 47% in 2007) [27] and Brazil (approximately 48% in 2010) [28], our findings suggested that the rate of sexually active female college students had gradually increased in China, ranging from approximately 8.6% in 2006 [12], to approximately 18% in 2010 [13], to approximately 20.6% in this study. Approximately 5.8% of female college students engaged in sexual intercourse after consuming alcohol, which was shown to be closely related to sexual behavior [13]. Approximately 6.1% of the students from rural areas had engaged in commercial sex during the last year, which was significantly higher than that of students from urban areas (approximately 3.5%). However, only 23.1% (rural: 19.3%; and urban: 24.5%) of these sexually active students almost always used a condom when having sexual intercourse with casual partners. Currently, there are several factors behind this social phenomenon in China. We hypothesized that financial hardship was the main reason driving students from rural areas to engage in commercial sex behavior because our study revealed that the living standard of the students from rural areas was significantly lower than that of the students from urban areas. In contrast, vanity and/or pursuing a high-quality material life might be a driving force for students from urban areas in China. The majority of the students were potentially influenced by recent cultures such as “part-time girlfriend with a sugar daddy,” which was a pervasive social problem in Southeast Asia, particularly in Japan [29]. Currently in China, a surprising number of female college students and even secondary school students regularly engage in commercial sex with older men for money in their spare time [12, 13, 18].

Approximately 6.7% of these students had multiple sexual partners during the last year, which was higher than the percentage found in a previous study in China (approximately 5.3% in 2009) [13]. Having multiple sex partners is one of the most important factors associated with the acquisition of HIV/STD infection [28]. Additionally, our survey found that the patterns of sexual intercourse among Chinese female college students had become diverse. Approximately 1.7% and 5.1% of the students had engaged in oral sex and anal sex without a condom, respectively, although the rates were lower than the rates in Western countries, such as Sweden (approximately 24% compared to 25%) [30]. Alarmingly, FCCSs (especially those from rural areas) appear to be extremely vulnerable to HIV/AIDS as a subgroup in China. We hope that our study attracts the attention of the Chinese government to establish more specific HIV/AIDS prevention policies for Chinese young women, including not only female sex workers but also female college students. Furthermore, the Chinese government should continue strengthening sex education and relevant studies such as the life-planning skills training (LPS) in all types of schools [31–33], which has been shown to be effective for HIV/AIDS control in China.

## 5. Conclusion

Sexual attitudes and behaviors have become increasingly open and active among FCCSs from both urban and rural areas. However, HIV/AIDS knowledge is insufficient in this population, particularly in those from rural areas. A considerable proportion of the students engaged in high-risk sexual behaviors, such as unprotected commercial sex (particularly those from rural areas), which presents a hidden challenge for HIV/AIDS control in China. The Chinese government should establish more specific HIV/AIDS prevention policies for young Chinese women and continue strengthening safe-sex education in the future.

## Figures and Tables

**Table 1:** Sociodemographic characteristics of female college students from urban and rural areas in China.

Variables	Students from urban areas (*n* = 2071)	Students from rural areas (*n* = 598)	*P* value
Mean age	20.4 ± 1.7	20.7 ± 1.8	<0.0001
Major			0.128
Social science	914 (44.1%, 914/2071)	243 (40.6%, 243/598)	
Natural science	1157 (55.9%, 1157/2071)	355 (59.4%, 355/598)	
Family style			0.010
Two-parent family	1800 (86.9%, 1800/2071)	495 (82.8%, 495/598)	
Single-parent or orphaned family	271 (13.1%, 271/2071)	103 (17.2%, 103/598)	
Average expenditure per month			<0.0001
<1000 RMB per month	1216 (58.7%, 1216/2071)	476 (79.6%, 476/598)	
1000–4000 RMB per month	820 (39.6%, 820/2071)	111 (18.6%, 111/598)	
>4000 RMB per month	35 (1.7%, 35/2071)	11 (1.8%, 11/598)	
Current status of the accommodation			<0.0001
In school dormitory	1930 (93.2%, 1930/2071)	531 (88.8%, 531/598)	
Off-campus apartment	141 (6.8%, 141/2071)	67 (11.2%, 67/598)	
Personal love status			<0.0001
Single	844 (40.8%, 844/2071)	304 (50.8%, 304/598)	
Being in love or once been in love	1227 (59.2%, 1227/2071)	294 (49.0%, 293/598)	
Has already experienced sex?			0.859
Yes	426 (20.6%, 426/2071)	125 (20.9%, 125/598)	
No	1645 (79.4%, 1645/2071)	473 (79.1%, 473/598)	
Grade			0.654
Freshmen	512 (24.7%, 512/2071)	136 (22.7%, 136/598)	
Sophomore	638 (30.8%, 638/2071)	202 (33.8%, 202/598)	
Junior	511 (24.7%, 511/2071)	135 (22.6%, 135/598)	
Senior	232 (11.2%, 232/2071)	67 (11.2%, 67/598)	
Graduate students	178 (8.6%, 178/2071)	58 (9.7%, 58/598)	

**Table 2:** Sexual awareness and attitude of female college students from urban and rural areas in China.

Variables	Students from urban areas (*n* = 2071)	Students from rural areas (*n* = 598)	*P* value
Time of sexual awareness			<0.001
<15 years old	334 (16.1%, 334/2071)	60 (10.0%, 60/598)	
>15 years old	1737 (83.9%, 1737/2071)	538 (90.0%, 538/598)	
Age of first exposure to pornographic media			0.072
<15 years old	218 (10.5%, 218/2071)	48 (8.0%, 48/598)	
>15 years old	1853 (89.5%, 1853/2071)	550 (91.9%, 550/598)	
Attitudes towards commercial sex			0.014
Disapprove	1526 (73.7%, 1526/2071)	472 (78.9%, 472/598)	
Approve and neutral	537 (25.9%, 537/2071)	126 (21.1%, 126/598)	
Attitudes towards homosexuality			<0.001
Disapprove	414 (19.9%, 414/2071)	183 (30.6%, 183/598)	
Approve and neutral	1651 (79.7%, 1651/2071)	415 (69.4%, 415/598)	
Attitudes to premarital sex			0.001
Disapprove	892 (43.1%, 892/2071)	303 (50.7%, 303/598)	
Approve and neutral	1176 (56.8%, 1176/2071)	295 (49.3%, 295/598)	
Attitudes to extramarital sex			0.475
Disapprove	1625 (78.5%, 1625/2071)	478 (79.9%, 478/598)	
Approve and neutral	443 (21.4%, 443/2071)	120 (20.1%, 120/598)	
Predict their probability of suffering from STDs			0.009
High risk	28 (1.4%, 28/2071)	15 (2.5%, 15/598)	
Medium risk	44 (2.1%, 44/2071)	22 (3.7%, 22/598)	
Low risk	399 (19.3%, 399/2071)	112 (18.7%, 112/598)	
Impossible	694 (33.5%, 694/2071)	168 (28.1%, 168/598)	
Never considered	905 (43.7%, 905/2071)	279 (46.7%, 279/598)	

STDs: sexually transmitted diseases, except HIV/AIDS.

**Table 3:** Sexual behaviors of female college students from urban and rural areas in China.

Variables	Students from urban areas (*n* = 2071)	Students from rural areas (*n* = 598)	*P* value
Age of sexual first masturbation			0.246
<15 years old	85 (4.1%, 85/2071)	35 (5.9%, 35/598)	
>15 years old	385 (18.6%, 385/2071)	122 (20.4%, 122/598)	
Age of sexual first intercourse			0.046
<15 years old	52 (2.5%, 52/2071)	24 (4.0%, 24/598)	
>15 years old	374 (18.1%, 374/2071)	101 (16.9%, 101/598)	
Sex after alcohol			0.851
Yes	122 (5.9%, 122/2071)	34 (5.7%, 34/598)	
Never	1949 (94.1%, 1949/2071)	564 (94.3%, 564/598)	
Contraceptive methods used at first sex			0.493
Condom	126 (6.1%, 126/2071)	31 (5.2%, 31/598)	
Others such as pills	227 (11.0%, 227/2071)	74 (12.4%, 74/598)	
No	71 (3.4%, 71/2071)	20 (3.3%, 20/598)	
Condom use at last sexual intercourse			<0.0001
Yes	188 (9.1%, 188/2071)	28 (4.7%, 28/598)	
No	238 (11.5%, 238/2071)	97 (16.2%, 97/598)	
The last sexual partner			<0.0001
Heterosexual	258 (12.5%, 258/2071)	44 (7.4%, 44/598)	
Homosexual and/or bisexual	97 (4.7%, 97/2071)	43 (7.2%, 43/598)	
Commercial	73 (3.5%, 73/2071)	37 (6.2%, 37/598)	
Condom use with regular sex partners			0.028
Almost always	88 (4.2%, 88/2071)	13 (2.2%, 13/598)	
Sometimes	282 (13.6%, 282/2071)	96 (16.1%, 96/598)	
Never	52 (2.5%, 52/2071)	15 (8.7%, 52/598)	
Condom use with casual sex partners			0.478
Almost always	74 (3.6%, 74/2071)	21 (3.5%, 21/598)	
Sometimes	199 (9.6%, 199/2071)	75 (12.5%, 75/598)	
Never	29 (1.4%, 29/2071)	13 (2.2%, 13/598)	
Once involved with prostitution			0.004
Yes	73 (3.5%, 73/2071)	37 (6.2%, 37/598)	
No	1998 (96.5%, 1998/2071)	561 (93.8%, 561/598)	
Number of sexual partners during the last year			0.605
None	1691 (81.7%, 1691/2071)	483 (80.8%, 483/598)	
1	244 (11.8%, 244/2071)	70 (11.7%, 70/598)	
≥2	134 (6.5%, 134/2071)	44 (7.4%, 44/598)	
Vaginal sex with condom			<0.0001
Yes	167 (8.1%, 167/2071)	23 (3.8%, 23/598)	
No	1904 (91.9%, 1904/2071)	573 (95.8%, 573/598)	
Vaginal sex without condom			0.765
Yes	145 (7.0%, 145/2071)	44 (7.4%, 44/598)	
No	1926 (93.0%, 1926/2071)	554 (92.6%, 554/598)	
Oral sex with condom			0.050
Yes	97 (4.7%, 97/2071)	40 (6.7%, 40/598)	
No	1974 (95.3%, 1974/2071)	558 (93.3%, 558/598)	
Oral sex without condom			0.360
Yes	105 (5.1%, 105/2071)	36 (6.0%, 36/598)	
No	1966 (94.9%, 1966/2071)	562 (93.4%, 562/598)	
Anal sex with condom			0.638
Yes	42 (2.0%, 42/2071)	14 (2.3%, 14/598)	
No	2029 (98.0%, 2029/2071)	584 (97.7%, 584/598)	
Anal sex without condom			0.296
Yes	35 (1.7%, 35/2071)	14 (2.3%, 14/598)	
No	2036 (98.3%, 2036/2071)	584 (97.7%, 584/598)	
Once being diagnosed as having STDs			0.006
Yes	156 (7.5%, 156/2071)	66 (11.0%, 66/598)	
No	1915 (92.5%, 1915/2071)	532 (89.0%, 532/598)	

STDs: sexually transmitted diseases, except HIV/AIDS.

**Table 4:** Knowledge of female Chinese college students concerning safe-sex practices from urban and rural areas in this study.

Variables	Students from urban areas (*n* = 2071)	Students from rural areas (*n* = 598)	*P* value
Recognition of all types of STDs			0.006
Correct	165 (8.0%, 165/2071)	28 (4.7%, 28/598)	
Incorrect	1906 (92.0%, 1906/2071)	570 (95.3%, 570/598)	
Recognition of all transmission modes of AIDS			0.002
Correct	630 (30.4%, 630/2071)	142 (23.7%, 142/598)	
Incorrect	1441 (69.6%, 1441/2071)	456 (76.3%, 456/598)	
Recognition of prevention methods of AIDS			0.677
Correct	853 (41.2%, 853/2071)	252 (42.1%, 252/598)	
Incorrect	1218 (58.8%, 1218/2071)	346 (57.9%, 346/598)	
Recognition of the significance of condom use			0.081
Correct	416 (20.1%, 416/2071)	101 (16.9%, 101/598)	
Incorrect	1655 (79.9%, 1655/2071)	497 (83.1%, 497/598)	
Sex educated before entering university			<0.0001
Yes	865 (41.8%, 865/2071)	134 (22.4%, 134/598)	
No	1206 (58.2%, 1206/2071)	464 (77.6%, 464/598)	

STDs: sexually transmitted diseases, except HIV/AIDS.
